# Application of AllerCatPro 2.0 for protein safety assessments of consumer products

**DOI:** 10.3389/falgy.2023.1209495

**Published:** 2023-07-11

**Authors:** Nora L. Krutz, Ian Kimber, Jason Winget, Minh N. Nguyen, Vachiranee Limviphuvadh, Sebastian Maurer-Stroh, Catherine Mahony, G. Frank Gerberick

**Affiliations:** ^1^ NV Procter & Gamble Services Company SA, Global Product Stewardship, Strombeek-Bever, Belgium; ^2^ Faculty of Biology, Medicine and Health, University of Manchester, Manchester, United Kingdom; ^3^ The Procter & Gamble Company, Mason, OH, United States; ^4^ Bioinformatics Institute, Agency for Science, Technology and Research (A*STAR), Singapore, Singapore; ^5^ IFCS Programme, Singapore Institute for Food and Biotechnology Innovation, Agency for Science, Technology and Research (A*STAR), Singapore, Singapore; ^6^ YLL School of Medicine and Department of Biological Sciences, National University of Singapore, Singapore, Singapore; ^7^ Procter & Gamble, Global Product Stewardship, Reading, United Kingdom; ^8^ GF3 Consultancy LLC, West Chester, OH, United States

**Keywords:** *in silico*, bioinformatics, protein allergenicity, IgE antibody, risk assessment, botanicals, natural substances

## Abstract

Foreign proteins are potentially immunogenic, and a proportion of these are able to induce immune responses that result in allergic sensitization. Subsequent exposure of sensitized subjects to the inducing protein can provoke a variety of allergic reactions that may be severe, or even fatal. It has therefore been recognized for some time that it is important to determine *a priori* whether a given protein has the potential to induce allergic responses in exposed subjects. For example, the need to assess whether transgene products expressed in genetically engineered crop plants have allergenic properties. This is not necessarily a straightforward exercise (as discussed elsewhere in this edition), but the task becomes even more challenging when there is a need to conduct an overall allergenicity safety assessment of complex mixtures of proteins in botanicals or other natural sources that are to be used in consumer products. This paper describes a new paradigm for the allergenicity safety assessment of proteins that is based on the use of AllerCatPro 2.0, a new version of a previously described web application model developed for the characterization of the allergenic potential of proteins. Operational aspects of AllerCatPro 2.0 are described with emphasis on the application of new features that provide improvements in the predictions of allergenic properties such as the identification of proteins with high allergenic concern. Furthermore, the paper provides a description of strategies of how AllerCatPro 2.0 can best be deployed as a screening tool for identifying suitable proteins as ingredients in consumer products as well as a tool, in conjunction with label-free proteomic analysis, for identifying and semiquantifying protein allergens in complex materials. Lastly, the paper discusses the steps that are recommended for formal allergenicity safety assessment of novel consumer products which contain proteins, including consideration and integration of predicted consumer exposure metrics. The article therefore provides a holistic perspective of the processes through which effective protein safety assessments can be made of potential allergenic hazards and risks associated with exposure to proteins in consumer products, with a particular focus on the use of AllerCatPro 2.0 for this purpose.

## 1. Introduction

Proteins from foreign species have the potential to induce immune responses in exposed subjects. It is not uncommon that such foreign proteins are able to provoke an immune response that results in allergic sensitization (1, 2). There remains an important need, for the purpose of consumer protection, to evaluate accurately the potential of individual proteins and of proteins in complex mixtures to induce allergic sensitization and allergic reactions in exposed subjects.

Various strategies for the assessment of the allergenic potential of proteins (and lack thereof) have been described including the use of animal models (3), characterization of physicochemical properties considered to be associated with allergenic potential, and sequence homology with known human allergens (4, 5).

Although there is no doubt that progress has been made, there remains a requirement for consistent safety assessment strategies. Previously there has been a particular focus on assessment of the allergenic potential of novel gene products in genetically modified crop plants (6–8) and industrial enzymes (9–12). In this review the focus is instead on approaches to the allergenicity safety assessment of proteins in botanicals and other natural substances that are used as ingredients in consumer products.

The emphasis here is a description of a paradigm for protein safety assessment with respect to allergenic activity that utilizes the recently described AllerCatPro 2.0 (https://allercatpro.bii.a-star.edu.sg/), an updated web application model for the characterization of the allergenic potential of proteins (13). This review paper describes the key steps required for an effective safety assessment of the allergenic potential of proteins in consumer products, and the integration of AllerCatPro 2.0 into that process.

## 2. How to use AllerCatPro 2.0 to aid protein safety assessments

**Figure 1:**
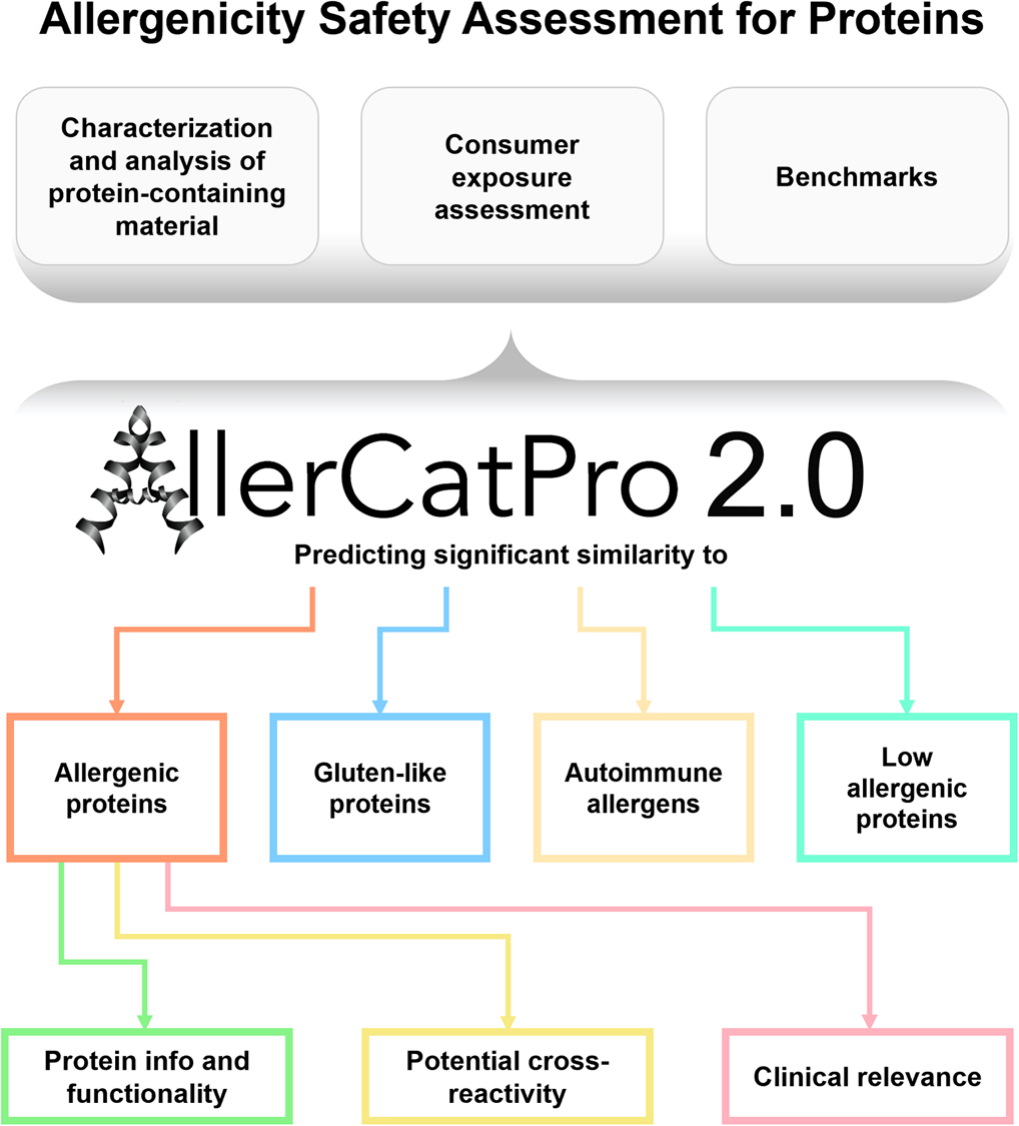
Summary of the features in AllerCatPro 2.0 which allow users to make informed decisions for the allergenicity safety assessment for proteins in consumer products.

AllerCatPro 2.0 provides information that allows users to make informed decisions (see Figure 1) for protein safety assessment. Deciding whether a protein is of concern and may cause IgE antibody-mediated allergies is the responsibility of the assessor and depends on many factors (including route of exposure, exposure concentration, etc.). The key steps of the risk assessment process are described in chapter 3. The first version of AllerCatPro (5) uniquely and conservatively predicts the level of evidence (strong, weak, and no evidence) for the allergenic potential of proteins based on the similarity in sequence and protein 3D structure with the most comprehensive dataset of proteins associated with allergenicity (including e.g., pollen and food allergens). With AllerCatPro 2.0 (13), several new features have been implemented that are unique among protein allergen prediction tools by providing information on potential cross-reactivity, protein functionality, clinical relevance, proteins associated with autoimmune diseases as well as similarity to proteins of low allergenic potential.

AllerCatPro 2.0 (13) builds upon a comprehensive dataset of protein allergens (*n* = 4,979) derived from the major databases FARRP (Food Allergy Research and Resource Program), COMPARE (Comprehensive Protein Allergen Resource), WHO/IUIS (World Health Organization/International Union of Immunological Societies), UniProtKB, and Allergome. Compared to AllerCatPro 1.7 (5), AllerCatPro 2.0 contains additional datasets of low allergenic proteins (*n* = 162), based on our previous publication (14), as well as human proteins associated with autoimmune diseases (*n* = 165) allowing separate predictions of similarity with each of the datasets. The users can input protein or nucleotide sequences in FASTA format. AllerCatPro 2.0 first screens the sequences for their similarity to Gluten-like proteins (“Gluten-like Q-repeats”), then for their similarity on a 3D structure level against a 3D structure database (*n* = 714 templates covering the majority of protein allergens), and finally, for similarity at the primary protein sequence level. In addition, AllerCatPro 2.0 identifies potential cross-reactivity with other protein allergens, protein information, and functionality, food and insect allergens, and information on clinical relevance.

A protein is predicted as having “strong evidence” or “weak evidence” for allergenicity if its 3D structure and/or sequence is significantly similar to a protein within the AllerCatPro 2.0 dataset (*n* = 4,979). The output gives a “Predicted most similar allergen” with a simplified allergen name (e.g., “Fel d 1” rather than “Fel d 1.0101”) along with the species name. Proteins are predicted with “no evidence” for allergenic potential if the protein sequence does not trigger a hit for similarity to known allergens on a 3D structure level or a sequence level above 35% sequence identity to known allergens within the 80-amino acid window as well as a sequence identity of at least 3 short hexamers with known allergens (3 × 6-mer sequence identity rule). Any similarity to proteins associated with autoimmune diseases or low allergenic potential is shown in separate columns with the percent identity values as a link to a different tab displaying a list and percent identity table, like the potential cross-reactivity table.

With the new features, AllerCatPro 2.0 can help with the identification of proteins of high allergenic concern (e.g., clinically significant food allergens) that would usually constitute thorough safety assessment for consumer products. These new features facilitate identifying and semi-quantifying source-specific allergens in complex materials when used in combination with label-free proteomics (15).

### 2.1. Features in AllerCatPro 2.0 for identifying proteins of high allergenic concern

The newly implemented features in AllerCatPro 2.0 (13) provide information to help assessors identify protein allergens of high allergenic concern. High allergenic concern needs to be determined by the assessor as it depends on the use of the protein-containing ingredient in consumer products. In the following, the features for cross-reactivity, protein families, allergen information and clinical relevance as well as the similarity to Gluten-like proteins are explained and illustrated with examples.

The potential cross-reactivity feature can provide additional information on whether the input protein is structurally similar to a large number of protein allergen sequences or, conversely, a unique or less well-characterized protein allergen. Identification of a large number of potential cross-reactive protein allergens may indicate that the input protein is similar to a specific protein sequence with many different variants in the AllerCatPro database or a structurally conserved protein family that is associated with allergenicity in different species, such as the prolamin superfamily (16) or even a novel protein family with only a few characterized protein allergens (17). The number of potential cross-reactive protein allergens appears in the column “potential cross-reactivity of query protein (# and links)” and provides a link to a new tab with the list of all significantly similar sequences in the AllerCatPro 2.0 database. If no information is shown in the column, then the input protein sequence is not significantly similar to an allergen in the AllerCatPro 2.0 database.

**Figure 2:**
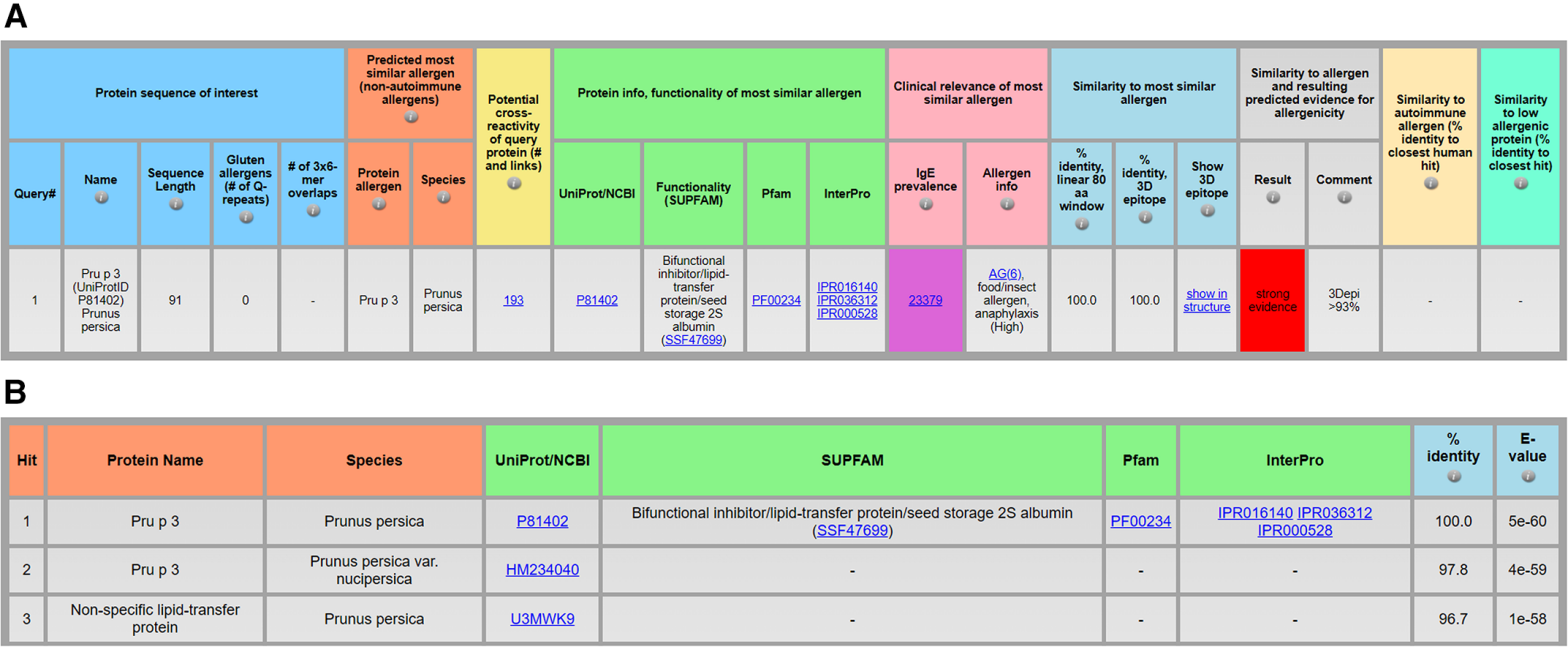
Identification of a protein allergen and potential cross-reactive allergens, exemplified by the protein sequence of Pru p 3 (UniProtID P81402) from *Prunus persica* (**A**) and the top 3 of 193 protein sequences predicted to be significantly similar to the query protein (**B**) using AllerCatPro 2.0.

Figure 2A shows results for Pru p 3 (UniProtID P81402, Supplement 1A), a non-specific lipid transfer protein (NLTP1) from peach that is often associated with cross-reactivity. AllerCatPro 2.0 identifies 193 potential cross-reactive protein sequences among its data set (*n* = 4,979) that are significantly similar to the input sequence. Among the 193 sequences there are non-specific lipid transfer proteins from other organisms, matching 28 of 46 UniProtIDs and 38 of 46 allergen names (Supplement 1B) of the WHO/IUIS list of non-specific lipid transfer proteins (18). As shown in Figure 2B, the table lists three cross-reactive protein sequences with their percent identity and the BLAST (Basic Local Alignment Search Tool) *E*-value (Expect value). The *E*-value indicates the probability due to chance, and thus, the closer the value is to zero, the more significant (but not random) the similarity towards the query protein sequence. Sequences are ranked from lowest to highest *E*-value and thus by significance (13).

Another new feature provides links for assessors to obtain protein information on the functionality of the predicted most similar known allergen. The protein family and functionality as well as physicochemical properties may contribute to clinically relevant allergenicity (16, 19). Therefore, links to UniProt or NCBI, Pfam, InterPro, and/or SUPFAM (Figure 2A) can help users to conveniently review information and download related protein sequences (e.g., the same protein superfamily via SUPFAM). Running all protein sequences within a protein family in AllerCatPro 2.0 can help to evaluate the phylogenetic distribution of known allergens with the same functionality.

**Figure 3:**
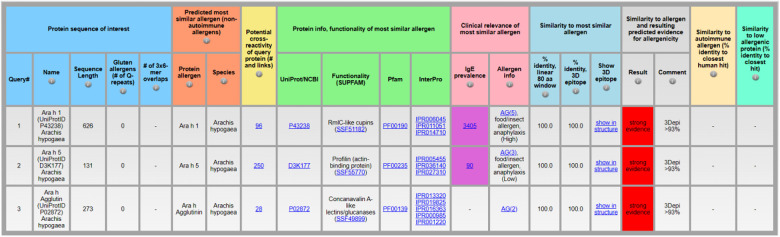
Identification of clinically relevant protein allergens, exemplified by the protein sequences of Ara h 1 (UniProtID P43238), Ara h 5 (UniProtID D3K177), and Ara h Agglutin (UniProtID P02872) from *Arachis hypogaea* using AllerCatPro 2.0.

The section “clinical relevance” contains the column for “IgE prevalence” (column shows the number individuals tested for specific IgE) and “allergen info” (Figure 3). While “allergen info” can help to identify known food or insect allergens, the number of individuals tested for specific IgE towards the protein allergen can help to evaluate prevalence via the link to the Allergome website that contains the list of published epidemiology studies and the corresponding number of individuals tested positive for specific IgE. As the numbers of individuals who tested positive for specific IgE vary significantly depending on the age and clinical history of the individuals tested (healthy, existing allergies) as well as geographic region and exposure opportunities to the protein allergen, it is difficult to estimate the overall IgE prevalence rate per protein allergen. Therefore, AllerCatPro 2.0 displays only the total number of individuals that have been tested for specific IgE towards the protein allergen in published epidemiology studies that are listed in Allergome as an indicator to differentiate between less and more well-studied allergens. The higher the number of tested individuals, the more likely the protein is a clinically relevant and frequently suspected allergen. However, a low number may still reflect clinical relevance for various reasons. For example, if the predicted most similar allergen is a variant or isoform of a well-characterized allergen but epidemiology data is not linked to the specific variant or isoform. Another possibility is a predicted most similar protein allergen that is not well investigated but belongs to a protein family commonly associated with allergens that are potentially cross-reactive. Protein allergens may also become clinically important and frequently suspected allergens for specific IgE testing but are currently too rare in the environment and/or exposure levels are too low to elicit allergic reactions.

As a demonstration, peanut allergens Ara h 1 (UniProtID P43238), Ara h 5 (UniProtID D3K177), and Ara h Agglutin (UniProtID P02872) show different numbers of individuals tested for specific IgE in the “IgE prevalence” column (Figure 3, input sequences in Supplement 2). For Ara h 1 the result is 3,405 individuals, for Ara h 5 it is 90, and for Ara h Agglutin no data is available to calculate the number of individuals tested for specific IgE towards the protein allergen. Via a click on the link embedded in the number, the AllerCatPro user can review more information in Allergome, including the number of individuals tested positive for specific IgE in different tested cohort groups and geographic regions, if available. In case of Ara h 5, the number is based on only 2 studies, one showing 13% frequency of specific IgE towards Ara h 5 among 40 individuals sensitized to peanut and with a history of symptoms and positive IgE test towards peanut allergens (20) and one study with 16% frequency of specific IgE among 50 individuals sensitized to peanut (21). Therefore, among peanut-sensitized individuals, Ara h 5 can be considered as statistically less clinically relevant compared to Ara h 1 and Ara h Agglutin does not seem to be significantly relevant.

Thus, the total number of tested individuals for specific IgE combined with the additional information and relevant publications on individuals tested positive for specific IgE towards the protein allergen that can be retrieved via the link to Allergome, can facilitate the assessment of whether a protein of interest is significantly similar to a well-characterized, clinically relevant protein allergen.

For the well-characterized protein allergens, the column “allergen info” provides an allergenicity score and a link to Allergome. The allergenicity score from Allergome reflects the current characterization status of the allergen based on availability of experimental data showing positive responses towards the allergen. Scores indicate the Allergome test types (Functional Test, Non-Functional Test, Skin Test, Conjunctival Provocation Test, Nasal Provocation Test, Bronchial Provocation Test, Oral Challenge, Epidemiology from Literature, ReTiME) and are shown as green, yellow, or red dots on the top of the Allergome website. In addition, specific protein allergens are indicated with “food/insect allergen” and “anaphylaxis (high)” if these protein allergens are typically associated with anaphylaxis (13). For example, the “allergen info” column for Ara h 1 (UniProtID P43238) indicates with the annotation “AG(5), food/insect allergen, anaphylaxis (High)” that it is a well-characterized protein allergen and has been associated with anaphylaxis among sensitized individuals, whereas Ara h 5 is less often associated with anaphylaxis (Figure 3).

Not all allergens with the potential to cause anaphylaxis and/or other severe allergic reactions such as hives, or respiratory symptoms are annotated as such in AllerCatPro 2.0 as there is no method to test all protein allergens for such effects systematically. Therefore, the absence of information should not be taken as evidence that a protein cannot cause severe allergic reactions.

With AllerCatPro 2.0, the user also can identify so-called Gluten-like Glutamine (Q)-repeats in protein sequences. The similarity to Gluten-like proteins is shown as a comment separately in addition to the sequence similarity comment to provide further information, whether the result is based on sequence similarity and/or Gluten-like prediction. For more information on how AllerCatPro identifies Gluten-like Q-repeats, see Maurer-Stroh et al. (5). In some cases, no similarity to a protein allergen is found in the AllerCatPro database, and the overall result “no evidence of allergenicity” is displayed despite a red-colored cell for “Gluten allergens (# of Q-repeats)” with the comment in the result section “Gluten-like Q-repeats”. This result means that the query protein has insignificant similarity to a known allergen but could be problematic for individuals with celiac disease in case of significant oral exposure. However, Q-repeats do not necessarily make a protein an allergen and do not anymore automatically result in “strong evidence” in AllerCatPro 2.0 (13). Assessors need to judge whether significant similarity to Gluten-like proteins for a given application should be considered as a potential issue and/or whether a specific benchmark should be used for the safety assessment. One example is if the protein were to be used in food or other products with significant oral exposure in which case Q-repeats may require further investigation and/or risk mitigation measures to ensure it is safe for individuals with celiac disease.

### 2.2. Guidance for using AllerCatPro 2.0 as a screening tool

The following approaches can be used as a practical guide to screen for suitable proteins as ingredients in consumer products using AllerCatPro 2.0. Usually, screening for a protein sequence is driven by a specific protein function of interest or to identify a suitable protein source. However, these screening approaches do not necessarily replace the protein safety assessment, as the presence of proteins as well as their relative abundance information in the material of interest are not within the scope of AllerCatPro 2.0. Instead, these techniques may help identify allergens of high concern and facilitate an early informed decision on whether the protein function and/or organism of interest is supportable for consumer products or whether risk mitigation measures (e.g., hydrolysis, see chapter 3, Step 1: Characterization and analysis of protein-containing materials) of the protein material should be considered.

In case a specific protein function is of interest for the consumer product, amino acid sequences annotated with desired functionality can be collected from InterPro and UniProt in FASTA (or nucleotide) format and processed with AllerCatPro 2.0 to evaluate according to different tiers:

Tier 1: Protein sequence is predicted with no evidence for allergenicity (and/or even high similarity to a protein with low allergenic potential), despite natural occurrence and abundance of the protein (family) in the environment. In case of oral uptake as a route of exposure, the protein sequence shows absence of similarity to Gluten-like proteins.
Tier 2: Protein sequence is predicted with weak or strong evidence for allergenicity, but with absence of information on potential IgE prevalence or a low number of individuals tested positive for specific IgE (compared to a high total number of tested individuals), despite natural occurrence and abundance of the protein (family) in the environment. Moreover, no annotation for “allergen info” and low similarity (including # of 3 × 6-mer overlaps, % identity, linear 80 aa window, and % identity, 3D epitope) to any known allergen (and/or even high similarity to a protein with low allergenic potential) as well as a low number of potential cross-reactive allergens. In case of oral uptake as a route of exposure, the protein sequence shows absence of similarity to Gluten-like proteins.

For both tiers 1 and 2, the assessor needs to evaluate whether the input sequence is a naturally occurring and abundant protein (family) in the environment to provide additional evidence for the absence of adverse effects despite opportunities for human exposure. A link in the AllerCatPro 2.0 output to Pfam, InterPro, and/or SUPFAM, if available, can provide information on the distribution of a protein. Readily available measured (semi-)quantitative protein abundance data is scarce, but can be found in e.g., “PaxDb”, the Protein Abundances Across Organisms (https://pax-db.org/) for some organisms (22).

**Figure 4:**
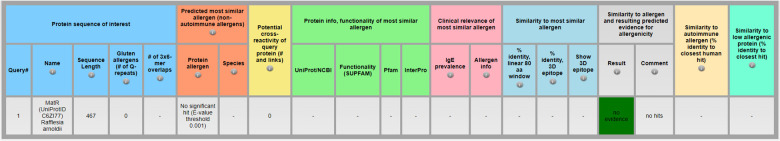
Importance of evaluating AllerCatPro 2.0 prediction results in context of information on human exposure, especially for less well characterized protein sequences using MatR (UniProtID C6ZI77) from *Rafflesia arnoldii* as an example in AllerCatPro 2.0.

If the input sequence is well characterized and from a protein source that is highly ubiquitous and abundant, then “no evidence for allergenicity” as output in AllerCatPro 2.0 can be indeed considered as likely low allergenic protein (14). In case of predicted high similarity to a known low allergenic protein, a benchmark related to the protein source of the predicted most similar low allergenic protein derived from occupational exposure and/or clinical data (23) may be appropriate to be used in the protein safety assessment. However, if an input sequence is from a protein source that is rare and where the likelihood of human exposure is very low, then a conservative benchmark (see chapter 3, Step 3: Benchmarks) would be advisable for the protein safety assessment, because the protein of interest could be outside the applicability domain of AllerCatPro 2.0. Figure 4 shows the AllerCatPro 2.0 result for MatR (UniProtID C6ZI77, input sequence in Supplement 3), a less well-characterized protein sequence from *Rafflesia arnoldii*, a rare plant endemic to the island of Sumatra in Indonesia. Although the AllerCatPro 2.0 prediction result shows no evidence for allergenicity, in absence of information on human exposure, the assessor may need to consider using a conservative benchmark for the safety assessment.

In case there is a need to screen a whole proteome to identify whether any source-specific protein allergens are to be expected in a material of interest, the protein sequences of the organism can be retrieved via UniProt and processed with AllerCatPro 2.0. However, it should be noted that this approach does not inform the user about the actual presence nor abundance of the protein sequences within a raw material of interest for the consumer product.

### 2.3. Identifying and semi-quantifying protein allergens within complex materials

AllerCatPro 2.0 in conjunction with label-free proteomic analysis by liquid chromatography–mass spectrometry (LC-MS) can be used to identify and semi-quantify proteins and potential allergens in complex mixtures. This approach has been used to identify proteins of low allergenic concern in highly abundant protein-containing materials (14) as well as to identify and semi-quantify source-specific protein allergens and potential cross-reactive allergens in complex mixtures (15). As part of a consumer safety assessment, this capability to identify and semi-quantify protein allergens can help to to determine a protein level of potential concern to calculate consumer exposure and compare results against the selected benchmark (see chapter 3, Step 3: Benchmarks).

In brief, the total protein content of the material of interest (derived by amino acid analysis, see Step 1: Characterization and analysis of protein-containing materials) is used along with a label-free proteomic analysis by LC-MS. If a high-quality proteome of the material of interest is available in UniProt, the identified and semi-quantified sequences from the proteomic analysis are used to run AllerCatPro 2.0. Most commonly, benchmarks such as eliciting dose values (24), are derived for protein sources, but not single protein allergens. Nevertheless, it is recommended to sum the relative abundances of all proteins with the same predicted most similar protein allergen to compare against the benchmark. Additionally, if many proteins are identified at very low relative abundance (e.g., below 0.001%) and predicted with “no evidence for allergenicity”, then it is recommended to exclude these sequences from the overall relative abundance calculation. Excluding these low abundance sequences increases the relative abundance of the most abundant proteins, which are more likely to drive the overall allergenicity potential of a complex mixture.

## 3. Protein safety assessment for consumer products

It is critical that any protein obtained from a natural source or botanical being introduced into a consumer product be thoroughly evaluated for its potential to induce or elicit an IgE-mediated immune response. There are historical examples of where protein in personal care products has led to the development of IgE antibodies and/or the development of allergic symptoms. Most examples are immune responses due to food protein allergens in consumer products. Therefore, knowing if the protein source is a known allergen found in food is essential.

One example of high prevalence of sensitized individuals with serious allergic reactions including anaphylaxis and contact urticaria resulting from the use of products containing this substance to the product as well as the food was the serious outbreak of allergic (including anaphylaxis) reactions in Japan due a facial soap containing 0.3% hydrolyzed wheat protein (25, 26). From 2009 to 2013, 1,900 cases had been reported (27) leading to many investigations to better understand underlying mechanisms of sensitization and the link to food allergies (28). To date, hydrolyzed wheat materials are commonly used in cosmetic products and are considered safe for consumers if the molecular weight average is ≤3.5 kDa (29).

Similar examples of potentially not fully hydrolyzed food allergens in consumer products leading to IgE-mediated allergic reactions involve skin products containing milk proteins. A soap product which contained goat milk and was used over years to treat dry skin and mild eczema led to sensitization in a woman who experienced an episode of goat cheese induced anaphylaxis (30). In another case study, a woman with a compromised skin barrier had been using a skin moisturizer containing goat milk for 4 months before she developed a generalized allergic reaction characterized by urticaria and rapidly evolving oral and upper airway angioedema immediately after eating goat cheese (31).

Besides food allergens, examples of allergic reactions to non-food protein allergens in consumer products include a quaternized hydrolyzed bovine collagen preparation (Crotein Q) in a hair care product that led to the development of allergic symptoms in hairdressers and consumers (32–34) and the use of a serine protease in a prototype bar soap and a body lotion (35, 36).

It is well established that skin contact with food allergens including peanut proteins can induce sensitization and the development of food allergy in particular when the skin barrier function is compromised (37). Skin contact with non-food enzymes capable of altering the epithelial barrier can enhance sensitization although levels and duration of exposure are determining factors, as evidenced by the fact that e.g., laundry and cleaning products do not appear to pose a risk for allergic reactions to enzymes (38). However, skin products for eczema patients are focused on individuals that may be more susceptible to sensitization via skin contact and thus at higher risk to develop severe allergic reactions to food allergens present in these skin products (39, 40). Therefore, it is important for assessors to understand the product use and the consumer practices and weigh uncertainties into the safety assessment. Mitigating the risk by labeling of consumer products is critical to inform consumers but may not be sufficient to fully protect sensitized individuals.

These examples of allergic reactions to consumer products highlight the need for methods and approaches for evaluating proteins for their allergenic potential. A tiered safety assessment approach addressing protein-containing botanicals and natural extracts has been published with emphasis that such materials can safely be used in cosmetic products when evaluated appropriately (41). There is evidence indicating that some proteins are more potent allergens based on what is known about their exposures and clinical outcomes, while other proteins are of less allergenic potential (14). However, no protein safety assessment is needed if there is no direct or indirect consumer exposure to proteins derived from product use, including potential product misuse. For example, no evaluation is necessary if the protein in the product is fully encapsulated and/or only released in an enclosed system. Consumer exposure to industrial enzymes in laundry and cleaning products can be considered of low concern based on low exposures supported by the evidence of absence of adverse effects from occupational medical surveillance programs as well as clinical data and information from product post-market surveillance (42, 43). If such surveillance data is not available for the protein of interest and consumer exposure cannot be excluded, then the first key step in the protein safety assessment is to evaluate if there is any protein in the material. The following proposes a step-by-step approach for evaluating the risk of inducing and eliciting an IgE-mediated allergic response to proteins in consumer products.

### 3.1. Step 1: characterization and analysis of protein-containing materials

Gathering information about the botanical or natural substance (e.g., host species name, part of species, extraction methods) is an important first step to understanding whether or not a more detailed safety assessment approach should be considered (44, 45). Generally, humans are exposed to a large amount of protein through the environment (pollen, dust) and food. However, when adding proteins to consumer products, the route, magnitude, duration, and frequency of exposure to the protein may change. Furthermore, the matrix of exposure is different than natural exposure to proteins, e.g., an irritant formulation to the skin or a product for individuals with eczema may facilitate the induction of sensitization (39, 40).

Nevertheless, many proteins, protein-containing botanicals, and natural substances have been safely used for many years, e.g., as traditional herbal medicinal plants and/or in cosmetic products. Information on protein use levels in cosmetic products for certain botanicals/natural substances can be retrieved from online databases such as Cosmetic Ingredient Review (https://www.cir-safety.org/). To screen whether any adverse allergic reactions to the material have already been reported, various databases can be queried (http://allergen.org/, https://www.allergome.org/, http://research.bmh.manchester.ac.uk/informall/allergenic-foods/, https://www.thermofisher.com/diagnostic-education/hcp/wo/en/resource-center/allergen-encyclopedia.html, etc.).

If needed, various methods with different strengths and limitations can measure total protein content. Standard protein assays, like bicinchoninic acid (BCA, Pierce) or Bradford reagent (Bio-Rad), may be useful in certain cases as a confirmatory method to demonstrate the absence of significant protein concentrations but are generally considered too variable in their performance to support a quantitative safety assessment. Other methods, such as the Kjeldahl Nitrogen method [approx. limit of detection (LOQ) protein: 31 ppm] or Chemiluminescent Nitrogen Detection (approx. LOQ protein: 1,875 ppm), may be appropriate in cases where either the total protein content in the material is not of high concern and the material is not considered to have high non-protein nitrogen sources (e.g., nitrogenous bases), as these would lead to an overestimation of the total protein content. If more precise protein quantification is needed, the sample's amino acid analysis (LOQ protein: 0.1 ppm) is recommended. In brief, amino acid analysis allows for the quantification of total amino acid content by amino acid separation and quantification after acid hydrolysis (46, 47).

Besides the total protein content, single protein allergens can be identified and quantified by the commonly used enzyme-linked immunosorbent assay (ELISA). However, this method relies on the availability of allergen-specific antibodies and the quantification of specific protein allergen targets only. Alternatively, proteins in complex materials (e.g., botanicals, natural substances) can be identified and semi-quantified for protein safety assessments using label-free proteomic analysis (14, 15). The determined material-specific protein level requiring a protein safety assessment can be used to calculate consumer exposure and to compare exposure results with benchmarks.

If there is protein in the material which is of high allergenic concern, but the protein is fully hydrolyzed, it is believed that using a cutoff for the length of peptides protects individuals from eliciting an IgE-mediated allergic response. Hydrolyzed protein results from chemical or enzymatic protein hydrolysis into amino acids and peptides of various sizes. Small polypeptides (less than 30 amino acids) are considered too small to cross-link two IgE antibodies, a required step to elicit IgE-mediated reactions (2, 48–50). A 3.5 kDa cutoff for protein exposure has been proposed based on molecular weight restrictions found for hydrolyzed wheat protein/gluten (51). Numerous studies in the literature report on the efficacy of denaturing known protein allergens using several approaches (52–55). However, it is important to note that other investigators have reported that allergenic proteins can be thermostable, or their activity can be increased with heat treatment (56–58).

In the above-mentioned example of a hydrolyzed wheat protein in a facial soap associated with anaphylaxis reactions in Japan, the wheat protein obtained from gluten was only partially hydrolyzed using hydrogen chloride at 95°C for 40 min. The molecular weight of the main band of hydrolyzed wheat protein, as determined with sodium dodecyl sulfate-polyacrylamide gel electrophoresis (SDS-PAGE), was 40–50 kDa (26, 59). In addition to wheat-derived material, peptide fragments from hydrolyzed milk protein material can preserve immunoreactive epitopes, which may explain the occasional occurrence of reactions to casein- or whey-based hydrolyzed formulas (60). The method and the intensity of hydrolysis determines the size of the generated fragments and their potential allergenicity (28). Thus, efficient methods to hydrolyze protein and confirm the size of peptides below the 3.5 kDa are important to mitigate the risk of potentially allergenic protein fragments within the material of interest. To add further conservatism to this approach, it has been proposed to use a more stringent cutoff of 2.5 kDa (41). If a successful process is used during manufacturing for the denaturation of proteins, the potential for eliciting and possibly also inducing an IgE-mediated response can be reduced significantly or even eliminated.

To confirm analytically the extent of hydrolysis of a protein-containing material, e.g., an SDS-PAGE can be used to provide information on the molecular weight distribution of the protein composition and the estimated concentration of protein fragments above the size threshold (e.g., 3.5 or 2.5 kDa) in the material. Using appropriate markers for the low protein size range and sensitive staining methods can help to decrease the limit for quantification and detection of proteins if needed. The resulting protein fragments above the cutoff in the material of interest can be used to calculate consumer exposure and compare exposure against an appropriate benchmark.

### 3.2. Step 2: consumer exposure assessment

If consumer exposure cannot be excluded, then, it is necessary to conduct an assessment to accurately assess the risk of inducing and eliciting IgE-mediated allergic reactions. Typical habits & practices data are useful to assess intended and foreseeable product uses. In evaluating the risk of inducing and eliciting an IgE-mediated allergic response, the assessor should address for all relevant routes of exposure the magnitude, duration, and frequency of exposure to the protein in the product. While the ConsExpo Fact Sheets (61) contain valuable information on calculating consumer exposure, many consumer exposure calculation tools (62) have been developed with a focus on consumer exposure to chemicals but not to proteins. If there is no consumer exposure information and/or if refinement is needed for a more realistic consumer exposure assessment, exposures can also be simulated and protein levels measured in, for example, air (63, 64).

For products with very low consumer exposure, a Threshold of Toxicological Concern (TTC) concept, as recently proposed for cosmetic ingredients (65), would be of great value and should be further explored for proteins. For products with significant consumer exposure, it is important to take into consideration all possible routes of exposure for the protein safety assessment of consumer products, including inhalation, oral uptake, skin and mucosal contact as in some circumstances, e.g., skin exposure to protein allergens can also result in sensitization (66–68). The type of exposure certainly has an impact on the amount of protein needed to sensitize and/or elicit an adverse event (69).

### 3.3. Step 3: benchmarks

While no exposure thresholds are widely accepted, clinical benchmark values described in this chapter can be used with caution. Depending on the route of exposure and the expected potency of the protein (as a single ingredient or in mixtures), appropriate benchmarks should be selected for the safety assessment. In the absence of widely accepted models for assessing proteins for their respiratory allergy potential, the availability of benchmarks becomes critical to conducting protein safety assessments to protect consumers from developing IgE-mediated allergy to protein-containing products. Experience from occupational health studies, and patterns of food allergy and hay fever, for instance (9, 11, 70, 71), suggests that there are differences in potency.

Unfortunately, limited studies inform risk assessors on “no effect levels” or “low effect levels” to proteins contained in such products and the challenge is that it is extremely difficult in most situations to establish with any certainty whether differences in the prevalence of allergy to proteins are attributable to variable exposure, differences in the inherent sensitizing potency of a single protein, or both. Most work has been done with protease enzymes that are used in the detergent industry as well as other studies, to better understand the allergenic potency of enzymes. Based on many historical studies as well as decades of experience, the detergent industry has successfully managed the safe use of enzymes both by workers at the manufacturing plants and consumers at home (72, 73). Safe exposure levels have been empirically established together with implementation of strict air monitoring and health surveillance in detergent factories (43, 74). The threshold limits proposed for enzyme protein in occupational and consumer settings are 60 ng/m^3^ and 15 ng/m^3^, respectively (75).

For evaluating the potency of individual detergent enzymes to aid in setting occupational exposure guidelines, both the mouse intranasal test (MINT) and guinea pig intratracheal (GPIT) model have been used in the past (9–11). In these studies, the bacterial serine protease Alcalase (Subtilisin B) has been used as a benchmark for evaluating the potency of other enzymes. Data from the MINT and GPIT showed that, based on specific antibody titers, the bacterial alpha-amylase Termamyl and a fungal exocellulase were more potent sensitizers, and a fungal alpha-amylase (Fungamyl) was less potent than the bacterial serine proteases Alcalase and Savinase (9, 11). These data show that when exposure to the protein allergens can be controlled, differences in the potency of individual proteins can be observed *in vivo* for the induction of respiratory sensitization.

Prospective clinical studies designed to assess the safety of a bar soap or body lotion containing a protease enzyme revealed that these types of repeated exposures can be sufficient to induce IgE antibodies in some of the study populations (35, 36). In the bar soap study, 4 of the 62 test subjects developed IgE antibodies to the enzyme after 4–6 months of use of the bar soap (35). Laboratory measures showed that the soap aerosolized in the shower with average exposure to the enzyme of 10.9 ng/m^3^. In the body lotion study, 3 of the 864 test subjects developed enzyme-specific IgE antibodies after 12–14 months of intermittent use of the lotion (36). Exposure assessments for the enzyme-containing body lotions were reported to lead to enzyme levels in the air of 0.25–0.5 ng/m^3^ (36). These prospective clinical studies showed that exposure to the enzyme from the use of a personal care product that led to enzyme aerosol levels in the shower greater than 0.1 ng/m^3^ were sufficient to sensitize test subjects. However, it is essential to point out that repeated skin or mucosal exposures, shower conditions, and potential skin irritation effects likely played a role in the sensitization to the enzyme. In the absence of other data for evaluating proteins in personal or consumer products, a benchmark of 0.1 ng/m^3^ was proposed to be used for risk assessment (23, 41). This 0.1 ng/m^3^ benchmark is comparable to the calculated consumer exposure value of 0.0067 ng/m^3^ enzyme from detergent dust, which is based on 0.27 µg of enzyme-detergent dust each time a powder laundry product is poured into a washing machine and deemed safe for consumers (76). It is also comparable to the study conducted with 289 atopic consumers using enzyme-containing detergent products for hand laundry and personal cleansing for at least two years, which resulted in no evidence of IgE sensitization towards the enzymes (amylase and protease) in the products. The highest measured exposure from the detergent granules and laundry bar used in the study was observed during hand washing with enzyme-containing granules, with values up to 0.18 ng/m^3^ protease and dermal exposure of 0.75 ng/cm^2^ skin (77).

The highest reported inhalation benchmark for a consumer product is based on data from a clinical study of a protease-containing spot cleaning product with a trigger spray device (63). The study demonstrated no adverse effects were observed over 6 months in a carefully monitored atopic population with approximate exposures of 15 ng/m^3^. Magnitudes above, the no effect protein exposure levels considered safe for plant-based materials range from less than 0.1 mg/m^3^ (e.g., latex protein) to greater than 100 mg/m^3^ (e.g., corn protein) (69).

In the case of inhalation, much has been learned from certain detergent enzymes and occupational exposure data. For dermal exposure as well as oral uptake of protein allergens, there is not much known about the level needed to induce IgE sensitization (69), but for certain food allergens, oral uptake threshold doses for elicitation have been derived from studies with previously sensitized subjects challenged with food allergies (24, 78, 79).

In summary, only a few threshold values are available that can be helpful as guidelines for assessing consumer products containing proteins. Generally, the more potent the allergen, the smaller the amount required to trigger immunological priming and sensitization. The challenge is that it is extremely difficult in most situations to establish with any certainty whether differences in the prevalence of allergy to proteins are attributable to variable exposure, differences in the inherent sensitizing potency of a single protein, or both. Unfortunately, there is much less certainty around establishing potency estimations for protein allergens for deriving useful benchmarks (69).

## 4. Discussion

Allergic sensitization and allergic disease resulting from exposure to proteins is an important and common health issue. It is essential, therefore, that every care should be taken to minimize the risks of allergic sensitization resulting from consumer exposure to food protein allergens, and to proteins contained within other products to which exposure via a relevant route might occur. As described in this article, a substantial body of literature has accumulated over the past 25 years describing the challenges posed by a need for a safety assessment approach to assess protein allergenicity, and the development of methods to address that need (2, 5–12, 14, 69, 80). The purpose of this review article has been to provide a more holistic perspective on the processes through which effective protein safety assessments can be made of potential allergenic hazards and risks associated with exposure to proteins in consumer products, with a particular focus on the application for this purpose of AllerCatPro 2.0. Key considerations are described, including the characterization of the protein-containing material, an understanding of the total protein content and the concentration of individual proteins in that product, knowledge of anticipated conditions, routes and levels of exposure, comparisons against relevant benchmarks where available, and application of AllerCatPro 2.0. This builds on the experience that has been gained previously with AllerCatPro for the characterization of allergenic proteins (14, 81–83).

A case is made here for a protein allergy safety assessment process that is based on the use of AllerCatPro 2.0. It must be acknowledged, however, that there remain several areas of uncertainty regarding the acquisition of sensitization to protein allergens and the elicitation of allergic reactions, and that resolution of some of these uncertainties may pave the way to further improvements in the safety assessment process. For instance, it is still not clear what properties, or combination of properties, confer on proteins the ability to stimulate the class of immune response that will result in sensitization (1, 2, 19, 84–86). In addition, the influence of factors such as glycosylation and plant lipids are uncertain, or at least variable (87, 88). Moreover, there remain uncertainties about the routes of exposure that favor the development of sensitization to proteins, and how the timing and route of exposure can influence whether sensitization or immunological tolerance is induced (89, 90). Another important consideration in the context of safety assessment is the impact of heating and processing on the sensitizing potential of allergenic proteins (91). All of these factors have, to a greater or lesser extent, the potential to impact significantly on the allergenicity of proteins and should ideally be considered as part of a holistic safety assessment process.

As our understanding of the important variables that influence the development of sensitization to proteins grows there will be opportunities to refine and improve the safety assessment of proteins in consumer products. Until then it is necessary to be vigilant about ensuring the methods currently available are deployed appropriately and interpreted carefully, and that known allergens are excluded from consumer products, or incorporated only at concentrations considered to be safe for any anticipated routes of exposure. In the meantime, however, the processes outlined in this article, and the use of AllerCatPro 2.0, provide a sound basis for safety assessment. Nevertheless, there are continued opportunities for refinements and improvements to the application. For instance, the number of protein allergens that inform AllerCatPro has increased significantly with the AllerCatPro 2.0 version incorporating information on 4,979 protein allergens (13), and this number will no doubt increase further. However, it must be acknowledged that currently this version excludes information on industrial enzymes (e.g., detergent enzymes, extremozymes) other than the sequences annotated as protein allergens in FARRP, COMPARE, WHO/IUIS, UniProtKB, and Allergome. Moreover, the prediction model is not yet equipped to determine the allergenic potential of engineered protein sequences such as polymers and peptides. Notwithstanding current limitations, it is argued that the paradigm described here provides a reliable route to effective safety assessment.
