# Supplementary material for: Application of AllerCatPro 2.0 for protein safety assessments of consumer products
Source: Front Allergy. 2023 Jul 11;4:1209495. doi: 10.3389/falgy.2023.1209495 (PMC10367106; doi:10.3389/falgy.2023.1209495)
Supplement: Supplementary file 1 [file Datasheet1.docx]

**Supplements**

Supplement 1A

Input protein sequence of Pru p 3 (UniProtID P81402) from *Prunus persica* used to identify 193 potential cross-reactive protein sequences in AllerCatPro 2.0.

> Pru p 3 (UniProtID P81402) Prunus persica

ITCGQVSSALAPCIPYVRGGGAVPPACCNGIRNVNNLARTTPDRQAACNCLKQLSASVPGVNPNNAAALPGKCGVHIPYKISASTNCATVK

Supplement 1B

List of 193 potentially cross-reactive protein allergens based on the predicted significant similarity to the query protein Pru p 3 (UniProtID P81402) from *Prunus persica* using AllerCatPro 2.0 and the match of 28 of 46 UniProtIDs and 38 of 46 unique allergen names with the WHO/IUIS list of non-specific lipid transfer proteins (Skypala et al., 2021).

|  |  |  |  |  |  | Matches with the WHO/IUIS list of non-specific lipid transfer proteins (Skypala et al., 2021) | |
| --- | --- | --- | --- | --- | --- | --- | --- |
| **Hit** | **Protein Name** | **Species** | **UniProt/NCBI** | **% Sequence identity** | **E-value** | **Allergen** | **UniProt** |
| 1 | Pru p 3 | *Prunus persica* | [P81402](https://www.uniprot.org/uniprot/P81402) | 100 | 5.00E-60 | Pru p 3 | P81402 |
| 2 | Pru p 3 | *Prunus persica var. nucipersica* | [HM234040](https://www.ncbi.nlm.nih.gov/protein/HM234040) | 97.8 | 4.00E-59 | Pru p 3 |  |
| 3 | Non-specific lipid-transfer protein | *Prunus persica* | [U3MWK9](https://www.uniprot.org/uniprot/U3MWK9) | 96.7 | 1.00E-58 |  |  |
| 4 | Pru p 3 | *Prunus persica* | [Q4VUZ0](https://www.uniprot.org/uniprot/Q4VUZ0) | 96.7 | 2.00E-58 | Pru p 3 |  |
| 5 | Pru p 3 | *Prunus persica* | [Q9LED1](https://www.uniprot.org/uniprot/Q9LED1) | 97.8 | 2.00E-58 | Pru p 3 |  |
| 6 | Pru p 3 | *Prunus persica* | [Q8H2B2](https://www.uniprot.org/uniprot/Q8H2B2) | 97.8 | 4.00E-57 | Pru p 3 |  |
| 7 | Pru ar 3 | *Prunus armeniaca* | [E7CLQ6](https://www.uniprot.org/uniprot/E7CLQ6) | 91.2 | 6.00E-55 | Pru ar 3 |  |
| 8 | Pru ar 3 | *Prunus armeniaca* | [P81651](https://www.uniprot.org/uniprot/P81651) | 91.2 | 2.00E-54 | Pru ar 3 | P81651 |
| 9 | Pru av 3 | *Prunus avium* | [E7CLQ2](https://www.uniprot.org/uniprot/E7CLQ2) | 89 | 9.00E-54 | Pru av 3 |  |
| 10 | Pru d 3 | *Prunus domestica* | [P82534](https://www.uniprot.org/uniprot/P82534) | 90.1 | 1.00E-53 | Pru d 3 | P82534 |
| 11 | Pru ar 3 | *Prunus armeniaca* | [E7CLQ7](https://www.uniprot.org/uniprot/E7CLQ7) | 89 | 2.00E-53 | Pru ar 3 |  |
| 12 | Pru av 3 | *Prunus avium* | [HM234043](https://www.ncbi.nlm.nih.gov/protein/HM234043) | 87.9 | 7.00E-53 | Pru av 3 |  |
| 13 | Pru av 3 | *Prunus avium* | [Q9M5X8](https://www.uniprot.org/uniprot/Q9M5X8) | 87.9 | 8.00E-53 | Pru av 3 | Q9M5X8 |
| 14 | Pru ar 3 | *Prunus armeniaca* | [E7CLQ5](https://www.uniprot.org/uniprot/E7CLQ5) | 85.7 | 4.00E-52 | Pru ar 3 |  |
| 15 | Mal d 3 | *Malus domestica* | [Q5J011](https://www.uniprot.org/uniprot/Q5J011) | 80.2 | 3.00E-49 | Mal d 3 |  |
| 16 | Mal d 3 | *Malus domestica* | [Q9M5X7](https://www.uniprot.org/uniprot/Q9M5X7) | 80.2 | 4.00E-49 | Mal d 3 |  |
| 17 | Mal d 3 | *Malus domestica* | [Q2V6D8](https://www.uniprot.org/uniprot/Q2V6D8) | 80.2 | 4.00E-49 | Mal d 3 |  |
| 18 | Mal d 3 | *Malus domestica* | [Q5GLH0](https://www.uniprot.org/uniprot/Q5GLH0) | 80.2 | 7.00E-49 | Mal d 3 |  |
| 19 | Pru ar 3 | *Prunus armeniaca* | [E7CLQ4](https://www.uniprot.org/uniprot/E7CLQ4) | 81.3 | 4.00E-48 | Pru ar 3 |  |
| 20 | Mal d 3 | *Malus domestica* | [Q5J009](https://www.uniprot.org/uniprot/Q5J009) | 78 | 5.00E-47 | Mal d 3 |  |
| 21 | Mal d 3 | *Malus domestica* | [Q5J000](https://www.uniprot.org/uniprot/Q5J000) | 78 | 6.00E-47 | Mal d 3 |  |
| 22 | Pyr c 3 | *Pyrus communis* | [Q9M5X6](https://www.uniprot.org/uniprot/Q9M5X6) | 78 | 6.00E-47 | Pyr c 3 | Q9M5X6 |
| 23 | Non-specific lipid-transfer protein | *Pyrus communis* | [G8DM17](https://www.uniprot.org/uniprot/G8DM17) | 79.1 | 2.00E-46 |  |  |
| 24 | Mal d 3 | *Malus domestica* | [Q5IZZ5](https://www.uniprot.org/uniprot/Q5IZZ5) | 76.9 | 2.00E-46 | Mal d 3 |  |
| 25 | Mal d 3 | *Malus domestica* | [Q5IZZ6](https://www.uniprot.org/uniprot/Q5IZZ6) | 76.9 | 4.00E-46 | Mal d 3 |  |
| 26 | Non-specific lipid-transfer protein | *Pyrus communis* | [G8DM20](https://www.uniprot.org/uniprot/G8DM20) | 73.6 | 5.00E-44 |  |  |
| 27 | Non-specific lipid-transfer protein | *Pyrus communis* | [G8DM19](https://www.uniprot.org/uniprot/G8DM19) | 76.9 | 9.00E-44 |  |  |
| 28 | Fra a 3 | *Fragaria ananassa* | [Q8VX12](https://www.uniprot.org/uniprot/Q8VX12) | 68.1 | 3.00E-42 | Fra a 3 | Q8VX12 |
| 29 | Fra a 3 | *Fragaria ananassa* | [Q4PLT8](https://www.uniprot.org/uniprot/Q4PLT8) | 68.1 | 4.00E-42 | Fra a 3 |  |
| 30 | Fra a 3 | *Fragaria ananassa* | [Q4PLU0](https://www.uniprot.org/uniprot/Q4PLU0) | 68.1 | 5.00E-42 | Fra a 3 |  |
| 31 | Fra a 3 | *Fragaria ananassa* | [Q4PLT6](https://www.uniprot.org/uniprot/Q4PLT6) | 68.1 | 5.00E-42 | Fra a 3 |  |
| 32 | Lup an 3 | *Lupinus angustifolius* | [A0A4P1RWD8](https://www.uniprot.org/uniprot/A0A4P1RWD8) | 68.1 | 3.00E-41 | Lup an 3 |  |
| 33 | Rub i 3 | *Rubus idaeus* | [Q0Z8V0](https://www.uniprot.org/uniprot/Q0Z8V0) | 68.1 | 8.00E-41 | Rub i 3 | Q0Z8V0 |
| 34 | Fra a 3 | *Fragaria ananassa* | [Q4PLT7](https://www.uniprot.org/uniprot/Q4PLT7) | 67 | 1.00E-40 | Fra a 3 |  |
| 35 | Fra a 3 | *Fragaria ananassa* | [Q4PLT9](https://www.uniprot.org/uniprot/Q4PLT9) | 65.9 | 2.00E-40 | Fra a 3 |  |
| 36 | Pha v 3 | *Phaseolus vulgaris* | [D3W147](https://www.uniprot.org/uniprot/D3W147) | 64.8 | 8.00E-40 | Pha v 3 |  |
| 37 | Ara h 9 | *Arachis hypogaea* | [B6CEX8](https://www.uniprot.org/uniprot/B6CEX8) | 68.1 | 1.00E-39 | Ara h 9 | B6CEX8 |
| 38 | Cit s 3 | *Citrus sinensis* | [Q8L5S8](https://www.uniprot.org/uniprot/Q8L5S8) | 65.9 | 2.00E-39 | Cit s 3 |  |
| 39 | Mor n 3 | *Morus nigra* | [P85894](https://www.uniprot.org/uniprot/P85894) | 68.1 | 2.00E-39 | Mor n 3 | P85894 |
| 40 | Ara h 9 | *Arachis hypogaea* | [B6CEX8](https://www.uniprot.org/uniprot/B6CEX8) | 68.1 | 4.00E-39 | Ara h 9 | B6CEX8 |
| 41 | Pun g 1 | *Punica granatum* | [A0A059STC4](https://www.uniprot.org/uniprot/A0A059STC4) | 66.3 | 4.00E-39 | Pun g 1 | A0A059STC4 |
| 42 | Cit s 3 | *Citrus sinensis* | [Q6EV47](https://www.uniprot.org/uniprot/Q6EV47) | 64.8 | 1.00E-38 | Cit s 3 |  |
| 43 | Ara h 9 | *Arachis hypogaea* | [B6CEX8](https://www.uniprot.org/uniprot/B6CEX8) | 67 | 2.00E-38 | Ara h 9 | B6CEX8 |
| 44 | Ara h 9 | *Arachis hypogaea* | [B6CEX8](https://www.uniprot.org/uniprot/B6CEX8) | 66.7 | 7.00E-38 | Ara h 9 | B6CEX8 |
| 45 | Cas s 8 | *Castanea sativa* | [F1AHA2](https://www.uniprot.org/uniprot/F1AHA2) | 62.6 | 7.00E-38 | Cas s 8 |  |
| 46 | Hev b 12 | *Hevea brasiliensis* | [Q8RYA8](https://www.uniprot.org/uniprot/Q8RYA8) | 65.2 | 8.00E-38 | Hev b 12 | Q8RYA8 |
| 47 | Pha v 3 | *Phaseolus vulgaris* | [D3W146](https://www.uniprot.org/uniprot/D3W146) | 62.2 | 1.00E-37 | Pha v 3 | D3W146 |
| 48 | Fra a 3 | *Fragaria ananassa* | [Q4PLT5](https://www.uniprot.org/uniprot/Q4PLT5) | 62.6 | 2.00E-37 | Fra a 3 |  |
| 49 | Ara h 9 | *Arachis hypogaea* | [B6CEX8](https://www.uniprot.org/uniprot/B6CEX8) | 65.9 | 3.00E-37 | Ara h 9 | B6CEX8 |
| 50 | Ara h 9 | *Arachis hypogaea* | [B6CEX8](https://www.uniprot.org/uniprot/B6CEX8) | 64.8 | 6.00E-37 | Ara h 9 | B6CEX8 |
| 51 | Pun g 1 | *Punica granatum* | [A0A059ST23](https://www.uniprot.org/uniprot/A0A059ST23) | 66.7 | 8.00E-37 | Pun g 1 |  |
| 52 | Vit v 1 | *Vitis vinifera* | [Q850K5](https://www.uniprot.org/uniprot/Q850K5) | 62.9 | 1.00E-36 | Vit v 1 | Q850K5 |
| 53 | Lac s 1 | *Lactuca sativa* | [XP_023731522](https://www.ncbi.nlm.nih.gov/protein/XP_023731522) | 61.5 | 2.00E-36 | Lac s 1 |  |
| 54 | Lac s 1 | *Lactuca sativa* | [A0A2J6KL39](https://www.uniprot.org/uniprot/A0A2J6KL39) | 61.5 | 2.00E-36 | Lac s 1 |  |
| 55 | Pun g 1 | *Punica granatum* | [A0A059SSZ0](https://www.uniprot.org/uniprot/A0A059SSZ0) | 63 | 2.00E-36 | Pun g 1 |  |
| 56 | Lac s 1 | *Lactuca sativa* | [A1E2H5](https://www.uniprot.org/uniprot/A1E2H5) | 63.7 | 4.00E-36 | Lac s 1 |  |
| 57 | Jug r 3 | *Juglans regia* | [C5H617](https://www.uniprot.org/uniprot/C5H617) | 63 | 5.00E-36 | Jug r 3 | C5H617 |
| 58 | Can s 3 | *Cannabis sativa* | [W0U0V5](https://www.uniprot.org/uniprot/W0U0V5) | 61.5 | 7.00E-36 | Can s 3 | W0U0V5 |
| 59 | Vit v 1 | *Vitis vinifera* | [Q2QCI7](https://www.uniprot.org/uniprot/Q2QCI7) | 60 | 7.00E-36 | Vit v 1 |  |
| 60 | Vit v 1 | *Vitis vinifera* | [Q850K6](https://www.uniprot.org/uniprot/Q850K6) | 60 | 7.00E-36 | Vit v 1 |  |
| 61 | Pla or 3 | *Platanus orientalis* | [A9YUH6](https://www.uniprot.org/uniprot/A9YUH6) | 60.4 | 9.00E-36 |  |  |
| 62 | Pla a 3 | *Platanus acerifolia* | [Q14K71](https://www.uniprot.org/uniprot/Q14K71) | 59.3 | 2.00E-35 | Pla a 3 |  |
| 63 | Zea m 14 | *Zea mays* | [Q2XX14](https://www.uniprot.org/uniprot/Q2XX14) | 62.6 | 2.00E-35 | Zea m 14 |  |
| 64 | Cor a 8 | *Corylus avellana* | [Q9ATH2](https://www.uniprot.org/uniprot/Q9ATH2) | 59.3 | 2.00E-35 | Cor a 8 | Q9ATH2 |
| 65 | Zea m 14 | *Zea mays* | [Q2XX22](https://www.uniprot.org/uniprot/Q2XX22) | 62.6 | 5.00E-35 | Zea m 14 |  |
| 66 | Zea m 14 | *Zea mays* | [Q2XX24](https://www.uniprot.org/uniprot/Q2XX24) | 62.6 | 5.00E-35 | Zea m 14 |  |
| 67 | Zea m 14 | *Zea mays* | [Q2XX19](https://www.uniprot.org/uniprot/Q2XX19) | 62.6 | 5.00E-35 | Zea m 14 |  |
| 68 | Zea m 14 | *Zea mays* | [Q2XX21](https://www.uniprot.org/uniprot/Q2XX21) | 62.6 | 5.00E-35 | Zea m 14 |  |
| 69 | Zea m 14 | *Zea mays* | [Q2XX16](https://www.uniprot.org/uniprot/Q2XX16) | 62.6 | 8.00E-35 | Zea m 14 |  |
| 70 | Zea m 14 | *Zea mays* | [P19656](https://www.uniprot.org/uniprot/P19656) | 62.9 | 1.00E-34 | Zea m 14 | P19656 |
| 71 | Ara h 9 | *Arachis hypogaea* | [B6CG41](https://www.uniprot.org/uniprot/B6CG41) | 61.5 | 1.00E-34 | Ara h 9 |  |
| 72 | Zea m 14 | *Zea mays* | [Q2XX15](https://www.uniprot.org/uniprot/Q2XX15) | 62.6 | 1.00E-34 | Zea m 14 |  |
| 73 | Zea m 14 | *Zea mays* | [Q2XX25](https://www.uniprot.org/uniprot/Q2XX25) | 61.5 | 2.00E-34 | Zea m 14 |  |
| 74 | Ara h 9 | *Arachis hypogaea* | [B6CEX8](https://www.uniprot.org/uniprot/B6CEX8) | 61.5 | 2.00E-34 | Ara h 9 | B6CEX8 |
| 75 | Zea m 14 | *Zea mays* | [Q2XX23](https://www.uniprot.org/uniprot/Q2XX23) | 61.5 | 2.00E-34 | Zea m 14 |  |
| 76 | Lup an 3 | *Lupinus angustifolius* | [A0A1J7GK90](https://www.uniprot.org/uniprot/A0A1J7GK90) | 60.7 | 3.00E-34 | Lup an 3 |  |
| 77 | Zea m 14 | *Zea mays* | [B6SGP7](https://www.uniprot.org/uniprot/B6SGP7) | 61.5 | 3.00E-34 | Zea m 14 |  |
| 78 | Zea m 14 | *Zea mays* | [Q2XX17](https://www.uniprot.org/uniprot/Q2XX17) | 61.5 | 5.00E-34 | Zea m 14 |  |
| 79 | Ara h 9 | *Arachis hypogaea* | [B6CEX8](https://www.uniprot.org/uniprot/B6CEX8) | 60.4 | 7.00E-34 | Ara h 9 | B6CEX8 |
| 80 | Ara h 9 | *Arachis hypogaea* | [B6CEX8](https://www.uniprot.org/uniprot/B6CEX8) | 60.4 | 7.00E-34 | Ara h 9 | B6CEX8 |
| 81 | Ara h 9 | *Arachis hypogaea* | [B6CEX8](https://www.uniprot.org/uniprot/B6CEX8) | 60.4 | 7.00E-34 | Ara h 9 | B6CEX8 |
| 82 | Ara h 9 | *Arachis hypogaea* | [B6CEX8](https://www.uniprot.org/uniprot/B6CEX8) | 60.4 | 8.00E-34 | Ara h 9 | B6CEX8 |
| 83 | Len c 3 | *Lens culinaris* | [A0AT29](https://www.uniprot.org/uniprot/A0AT29) | 60.4 | 2.00E-33 | Len c 3 | A0AT29 |
| 84 | Ara h 9 | *Arachis hypogaea* | [B6CEX8](https://www.uniprot.org/uniprot/B6CEX8) | 59.3 | 3.00E-33 | Ara h 9 | B6CEX8 |
| 85 | Ara h 9 | *Arachis hypogaea* | [B6CEX8](https://www.uniprot.org/uniprot/B6CEX8) | 60.4 | 3.00E-33 | Ara h 9 | B6CEX8 |
| 86 | Ara h 9 | *Arachis hypogaea* | [B6CEX8](https://www.uniprot.org/uniprot/B6CEX8) | 59.3 | 3.00E-33 | Ara h 9 | B6CEX8 |
| 87 | Ara h 17 | *Arachis hypogaea* | [A0A510A9S3](https://www.uniprot.org/uniprot/A0A510A9S3) | 57.6 | 4.00E-33 | Ara h 17 |  |
| 88 | Ory s 14 | *Oryza sativa* | [A2ZDR8](https://www.uniprot.org/uniprot/A2ZDR8) | 57.6 | 4.00E-33 |  |  |
| 89 | Ara h 17 | *Arachis hypogaea* | [A0A445AL51](https://www.uniprot.org/uniprot/A0A445AL51) | 57.6 | 9.00E-33 | Ara h 17 |  |
| 90 | Zea m 14 | *Zea mays* | [B8QW34](https://www.uniprot.org/uniprot/B8QW34) | 59.3 | 9.00E-33 | Zea m 14 |  |
| 91 | Ara h 9 | *Arachis hypogaea* | [B6CEX8](https://www.uniprot.org/uniprot/B6CEX8) | 59.3 | 1.00E-32 | Ara h 9 | B6CEX8 |
| 92 | Lac s 1 | *Lactuca sativa* | [A1E2H4](https://www.uniprot.org/uniprot/A1E2H4) | 57.1 | 5.00E-32 | Lac s 1 |  |
| 93 | Ory s 14 | *Oryza sativa* | [O22482](https://www.uniprot.org/uniprot/O22482) | 55.6 | 2.00E-31 |  |  |
| 94 | Pru du 3 | *Prunus dulcis* | [C0L0I5](https://www.uniprot.org/uniprot/C0L0I5) | 58.1 | 2.00E-31 | Pru du 3 | C0L0I5 |
| 95 | Ara t 3 | *Arabidopsis thaliana* | [Q42589](https://www.uniprot.org/uniprot/Q42589) | 55.4 | 2.00E-31 |  |  |
| 96 | Ory s 14 | *Oryza sativa* | [Q7XJ39](https://www.uniprot.org/uniprot/Q7XJ39) | 54.4 | 2.00E-31 |  |  |
| 97 | Ory s 14 | *Oryza sativa* | [A3C7Z3](https://www.uniprot.org/uniprot/A3C7Z3) | 54.4 | 2.00E-31 |  |  |
| 98 | Ory s 14 | *Oryza sativa* | [A2ZAT0](https://www.uniprot.org/uniprot/A2ZAT0) | 54.4 | 2.00E-31 |  |  |
| 99 | Ory s 14 | *Oryza sativa* | [Q2QYL2](https://www.uniprot.org/uniprot/Q2QYL2) | 54.4 | 3.00E-31 |  |  |
| 100 | Non-specific lipid-transfer protein | *Platanus acerifolia* | [A0A0N9E6M0](https://www.uniprot.org/uniprot/A0A0N9E6M0) | 52.7 | 3.00E-31 |  |  |
| 101 | Zea m 14 | *Zea mays* | [B8QW37](https://www.uniprot.org/uniprot/B8QW37) | 58.4 | 3.00E-31 | Zea m 14 |  |
| 102 | Zea m 14 | *Zea mays* | [B8QW29](https://www.uniprot.org/uniprot/B8QW29) | 58.4 | 3.00E-31 | Zea m 14 |  |
| 103 | Zea m 14 | *Zea mays* | [B8QW53](https://www.uniprot.org/uniprot/B8QW53) | 58.4 | 4.00E-31 | Zea m 14 |  |
| 104 | Art gm 3 | *Artemisia gmelinii* | [ANC85022](https://www.ncbi.nlm.nih.gov/protein/ANC85022) | 51.6 | 5.00E-31 |  |  |
| 105 | Zea m 14 | *Zea mays* | [B8QW95](https://www.uniprot.org/uniprot/B8QW95) | 57.1 | 5.00E-31 | Zea m 14 |  |
| 106 | Zea m 14 | *Zea mays* | [Q2XX47](https://www.uniprot.org/uniprot/Q2XX47) | 58.6 | 6.00E-31 | Zea m 14 |  |
| 107 | Zea m 14 | *Zea mays* | [B8QW56](https://www.uniprot.org/uniprot/B8QW56) | 57.3 | 8.00E-31 | Zea m 14 |  |
| 108 | Art v 3 | *Artemisia vulgaris* | [C4MGH0](https://www.uniprot.org/uniprot/C4MGH0) | 51.6 | 8.00E-31 | Art v 3 |  |
| 109 | Zea m 14 | *Zea mays subsp. parviglumis* | [B8QW58](https://www.uniprot.org/uniprot/B8QW58) | 58.4 | 8.00E-31 | Zea m 14 |  |
| 110 | Pis s 3 | *Pisum sativum* | [2N81](https://www.ncbi.nlm.nih.gov/protein/2N81) | 58.1 | 1.00E-30 |  |  |
| 111 | Zea m 14 | *Zea mays* | [B8QW40](https://www.uniprot.org/uniprot/B8QW40) | 57.3 | 1.00E-30 | Zea m 14 |  |
| 112 | Api g 2 | *Apium graveolens* | [E6Y8S8](https://www.uniprot.org/uniprot/E6Y8S8) | 54.4 | 1.00E-30 | Api g 2 | E6Y8S8 |
| 113 | Pis s 3 | *Pisum sativum* | [A0A161AT60](https://www.uniprot.org/uniprot/A0A161AT60) | 57 | 1.00E-30 |  |  |
| 114 | Zea m 14 | *Zea mays* | [B6T089](https://www.uniprot.org/uniprot/B6T089) | 57.3 | 2.00E-30 | Zea m 14 |  |
| 115 | Non-specific lipid-transfer protein | *Brassica oleracea* | [A0A0D3B3Z0](https://www.uniprot.org/uniprot/A0A0D3B3Z0) | 58.2 | 2.00E-30 |  |  |
| 116 | Zea m 14 | *Zea mays* | [B6SY96](https://www.uniprot.org/uniprot/B6SY96) | 58.6 | 3.00E-30 | Zea m 14 |  |
| 117 | Non-specific lipid-transfer protein | *Vitis sp.* | [P80273](https://www.uniprot.org/uniprot/P80273) | 55.6 | 4.00E-30 |  |  |
| 118 | Art ca 3 | *Artemisia capillaris* | [ANC85021](https://www.ncbi.nlm.nih.gov/protein/ANC85021) | 50.5 | 5.00E-30 |  |  |
| 119 | Art an 3 | *Artemisia annua* | [ANC85017](https://www.ncbi.nlm.nih.gov/protein/ANC85017) | 50.5 | 5.00E-30 |  |  |
| 120 | Cro s 3 | *Crocus sativus* | [D2T0A5](https://www.uniprot.org/uniprot/D2T0A5) | 51.1 | 6.00E-30 |  |  |
| 121 | Ory s 14 | *Oryza sativa* | [Q0IQK9](https://www.uniprot.org/uniprot/Q0IQK9) | 53.3 | 7.00E-30 |  |  |
| 122 | Ory s 14 | *Oryza sativa* | [B7SDG3](https://www.uniprot.org/uniprot/B7SDG3) | 53.3 | 7.00E-30 |  |  |
| 123 | Ory s 14 | *Oryza sativa* | [A2ZHF1](https://www.uniprot.org/uniprot/A2ZHF1) | 53.3 | 7.00E-30 |  |  |
| 124 | Pis s 3 | *Pisum sativum* | [A0A158V755](https://www.uniprot.org/uniprot/A0A158V755) | 57 | 8.00E-30 |  |  |
| 125 | Art v 3 | *Artemisia vulgaris* | [C4MGG9](https://www.uniprot.org/uniprot/C4MGG9) | 49.5 | 1.00E-29 | Art v 3 |  |
| 126 | Art la 3 | *Artemisia lavandulifolia* | [ANC85024](https://www.ncbi.nlm.nih.gov/protein/ANC85024) | 49.5 | 1.00E-29 |  |  |
| 127 | Art ar 3 | *Artemisia argyi* | [ANC85019](https://www.ncbi.nlm.nih.gov/protein/ANC85019) | 49.5 | 1.00E-29 |  |  |
| 128 | Zea m 14 | *Zea mays* | [B8QW75](https://www.uniprot.org/uniprot/B8QW75) | 56.2 | 1.00E-29 | Zea m 14 |  |
| 129 | Ory s 14 | *Oryza sativa* | [Q2RBD2](https://www.uniprot.org/uniprot/Q2RBD2) | 52.2 | 2.00E-29 |  |  |
| 130 | Ory s 14 | *Oryza sativa* | [Q7XBA6](https://www.uniprot.org/uniprot/Q7XBA6) | 52.2 | 2.00E-29 |  |  |
| 131 | Ory s 14 | *Oryza sativa* | [A2ZAT1](https://www.uniprot.org/uniprot/A2ZAT1) | 52.2 | 2.00E-29 |  |  |
| 132 | Tri td 14 | *Triticum turgidum subsp. durum* | [Q5NE26](https://www.uniprot.org/uniprot/Q5NE26) | 52.2 | 3.00E-29 |  |  |
| 133 | Tri tu 14 | *Triticum turgidum* | [F6MEX1](https://www.uniprot.org/uniprot/F6MEX1) | 52.2 | 5.00E-29 | Tri tu 14 |  |
| 134 | Ory s 14 | *Oryza sativa* | [O22485](https://www.uniprot.org/uniprot/O22485) | 52.2 | 9.00E-29 |  |  |
| 135 | Art v 3 | *Artemisia vulgaris* | [C4MGH1](https://www.uniprot.org/uniprot/C4MGH1) | 46.2 | 1.00E-28 | Art v 3 |  |
| 136 | Dau c 3 | *Daucus carota* | [P27631](https://www.uniprot.org/uniprot/P27631) | 56.2 | 2.00E-28 |  |  |
| 137 | Pru p 3 | *Prunus persica* | [B6CQU7](https://www.uniprot.org/uniprot/B6CQU7) | 54.9 | 2.00E-28 | Pru p 3 |  |
| 138 | Art v 3 | *Artemisia vulgaris* | [C4MGH2](https://www.uniprot.org/uniprot/C4MGH2) | 46.2 | 3.00E-28 | Art v 3 |  |
| 139 | Pru av 3 | *Prunus avium* | [E7CLQ1](https://www.uniprot.org/uniprot/E7CLQ1) | 70.3 | 1.00E-27 | Pru av 3 |  |
| 140 | Sola l 3 | *Solanum lycopersicum* | [P93224](https://www.uniprot.org/uniprot/P93224) | 49.5 | 2.00E-27 | Sola l 3 | P93224 |
| 141 | Sola l 3 | *Solanum lycopersicum* | [Q4A1N0](https://www.uniprot.org/uniprot/Q4A1N0) | 49.5 | 2.00E-27 | Sola l 3 |  |
| 142 | Zea m 14 | *Zea mays* | [B6TTP1](https://www.uniprot.org/uniprot/B6TTP1) | 61.5 | 2.00E-27 | Zea m 14 |  |
| 143 | Art si 3 | *Artemisia sieversiana* | [ANC85026](https://www.ncbi.nlm.nih.gov/protein/ANC85026) | 47.3 | 2.00E-27 |  |  |
| 144 | Sin a 3 | *Sinapis alba* | [E6Y2L9](https://www.uniprot.org/uniprot/E6Y2L9) | 54.9 | 3.00E-27 | Sin a 3 | E6Y2L9 |
| 145 | Pru p 3 | *Prunus persica* | [B6CQU6](https://www.uniprot.org/uniprot/B6CQU6) | 53.8 | 3.00E-27 | Pru p 3 |  |
| 146 | Sola l 3 | *Solanum lycopersicum* | [Q4A1N1](https://www.uniprot.org/uniprot/Q4A1N1) | 52.7 | 3.00E-27 | Sola l 3 |  |
| 147 | Sola l 3 | *Solanum lycopersicum* | [P27056](https://www.uniprot.org/uniprot/P27056) | 49.5 | 3.00E-27 | Sola l 3 |  |
| 148 | Ara t 3 | *Arabidopsis thaliana* | [Q9S7I3](https://www.uniprot.org/uniprot/Q9S7I3) | 53.3 | 6.00E-27 |  |  |
| 149 | Ory s 14 | *Oryza sativa* | [Q0IQL2](https://www.uniprot.org/uniprot/Q0IQL2) | 51.6 | 2.00E-26 |  |  |
| 150 | Ory s 14 | *Oryza sativa* | [O65091](https://www.uniprot.org/uniprot/O65091) | 51.6 | 2.00E-26 |  |  |
| 151 | Non-specific lipid-transfer protein | *Hordeum vulgare* | [P07597](https://www.uniprot.org/uniprot/P07597) | 44.4 | 8.00E-26 |  |  |
| 152 | Tri a 14 | *Triticum aestivum* | [Q8GZB0](https://www.uniprot.org/uniprot/Q8GZB0) | 45.6 | 9.00E-26 | Tri a 14 |  |
| 153 | LTP 1 | *Hordeum vulgare* | [CAA42832](https://www.ncbi.nlm.nih.gov/protein/CAA42832) | 45.5 | 1.00E-25 |  |  |
| 154 | Tri a 14 | *Triticum aestivum* | [Q5NE27](https://www.uniprot.org/uniprot/Q5NE27) | 44.4 | 6.00E-25 | Tri a 14 |  |
| 155 | Act c 10 | *Actinidia chinensis* | [P85204](https://www.uniprot.org/uniprot/P85204) | 45.5 | 3.00E-24 |  |  |
| 156 | Act d 10 | *Actinidia deliciosa* | [P85206](https://www.uniprot.org/uniprot/P85206) | 47.3 | 6.00E-24 |  |  |
| 157 | Act d 10 | *Actinidia deliciosa* | [P85205](https://www.uniprot.org/uniprot/P85205) | 47.3 | 7.00E-24 |  |  |
| 158 | Sola l 7 | *Solanum lycopersicum* | [K4AYX7](https://www.uniprot.org/uniprot/K4AYX7) | 42.2 | 2.00E-22 |  |  |
| 159 | Cro s 3 | *Crocus sativus* | [D2T0A6](https://www.uniprot.org/uniprot/D2T0A6) | 43.5 | 2.00E-22 |  |  |
| 160 | Non-specific lipid-transfer protein | *Pisum sativum* | [A0A158V976](https://www.uniprot.org/uniprot/A0A158V976) | 51.6 | 7.00E-22 |  |  |
| 161 | Bra o 3 | *Brassica oleracea* | [Q39382](https://www.uniprot.org/uniprot/Q39382) | 47.7 | 1.00E-21 | Bra o 3 |  |
| 162 | Tri a 14 | *Triticum aestivum* | [D2T2K2](https://www.uniprot.org/uniprot/D2T2K2) | 46.7 | 2.00E-20 | Tri a 14 | D2T2K2 |
| 163 | Tri a 14 | *Triticum aestivum* | [D2T2K0](https://www.uniprot.org/uniprot/D2T2K0) | 45.7 | 2.00E-20 | Tri a 14 |  |
| 164 | Hor v 14 | *Hordeum vulgare subsp. vulgare* | [F2CY84](https://www.uniprot.org/uniprot/F2CY84) | 44.6 | 5.00E-20 |  |  |
| 165 | Ory s 14 | *Oryza sativa* | [Q2QYL3](https://www.uniprot.org/uniprot/Q2QYL3) | 41.1 | 4.00E-19 |  |  |
| 166 | Ory s 14 | *Oryza sativa* | [B7E4W9](https://www.uniprot.org/uniprot/B7E4W9) | 41.1 | 4.00E-19 |  |  |
| 167 | Ory s 14 | *Oryza sativa* | [A2ZAS9](https://www.uniprot.org/uniprot/A2ZAS9) | 41.1 | 4.00E-19 |  |  |
| 168 | Tri a 14 | *Triticum aestivum* | [D2T2K1](https://www.uniprot.org/uniprot/D2T2K1) | 43.5 | 5.00E-19 | Tri a 14 |  |
| 169 | Hel a 3 | *Helianthus annuus* | [P82007](https://www.uniprot.org/uniprot/P82007) | 42.2 | 6.00E-19 | Hel a 3 |  |
| 170 | Tri a 14 | *Triticum aestivum* | [Q2PCB7](https://www.uniprot.org/uniprot/Q2PCB7) | 43.5 | 2.00E-18 | Tri a 14 |  |
| 171 | Tri a 14 | *Triticum aestivum* | [Q2PCD2](https://www.uniprot.org/uniprot/Q2PCD2) | 43.5 | 2.00E-18 | Tri a 14 |  |
| 172 | Tri a 14 | *Triticum aestivum* | [Q2PCD1](https://www.uniprot.org/uniprot/Q2PCD1) | 43.5 | 2.00E-18 | Tri a 14 |  |
| 173 | Tri a 14 | *Triticum aestivum* | [Q2PCB8](https://www.uniprot.org/uniprot/Q2PCB8) | 42.4 | 7.00E-18 | Tri a 14 |  |
| 174 | Tri a 14 | *Triticum aestivum* | [Q5NE31](https://www.uniprot.org/uniprot/Q5NE31) | 43.5 | 2.00E-17 | Tri a 14 |  |
| 175 | Amb a 6 | *Ambrosia artemisiifolia* | [O04004](https://www.uniprot.org/uniprot/O04004) | 41.3 | 1.00E-12 | Amb a 6 | O04004 |
| 176 | Par j 2 | *Parietaria judaica* | [O04403](https://www.uniprot.org/uniprot/O04403) | 27.5 | 8.00E-12 | Par j 2 |  |
| 177 | Par j 2 | *Parietaria judaica* | [P55958](https://www.uniprot.org/uniprot/P55958) | 26.4 | 8.00E-12 | Par j 2 | P55958 |
| 178 | Par j 1 | *Parietaria judaica* | [Q40905](https://www.uniprot.org/uniprot/Q40905) | 32 | 6.00E-11 | Par j 1 |  |
| 179 | Par j 1 | *Parietaria judaica* | [P43217](https://www.uniprot.org/uniprot/P43217) | 30.1 | 2.00E-10 | Par j 1 | P43217 |
| 180 | Par j 1 | *Parietaria judaica* | [Q1JTN5](https://www.uniprot.org/uniprot/Q1JTN5) | 30.7 | 3.00E-10 | Par j 1 |  |
| 181 | Non-specific lipid-transfer protein | *Vitis sp.* | [P33556](https://www.uniprot.org/uniprot/P33556) | 61.1 | 9.00E-10 |  |  |
| 182 | Lyc ba 3 | *Lycium barbarum* | [B3A0N2](https://www.uniprot.org/uniprot/B3A0N2) | 57.5 | 1.00E-09 |  |  |
| 183 | Par j 1 | *Parietaria judaica* | [O04404](https://www.uniprot.org/uniprot/O04404) | 30.7 | 4.00E-09 | Par j 1 |  |
| 184 | Ara h 9 | *Arachis hypogaea* | [B6CEX8](https://www.uniprot.org/uniprot/B6CEX8) | 42.6 | 1.00E-08 | Ara h 9 | B6CEX8 |
| 185 | Par j I | *Parietaria judaica* | [2008179A](https://www.ncbi.nlm.nih.gov/protein/2008179A) | 29.1 | 2.00E-08 |  |  |
| 186 | Jug r 3 | *Juglans regia* | [C5H617](https://www.uniprot.org/uniprot/C5H617) | 59.4 | 6.00E-08 | Jug r 3 | C5H617 |
| 187 | Art v 3 | *Artemisia vulgaris* | [P0C088](https://www.uniprot.org/uniprot/P0C088) | 41.7 | 9.00E-08 | Art v 3 | P0C088 |
| 188 | Vit v 1 | *Vitis sp.* | [P80274](https://www.uniprot.org/uniprot/P80274) | 60.7 | 1.00E-06 | Vit v 1 |  |
| 189 | Ara h 9 | *Arachis hypogaea* | [B6CEX8](https://www.uniprot.org/uniprot/B6CEX8) | 43.5 | 5.00E-06 | Ara h 9 | B6CEX8 |
| 190 | Cas s 8 | *Castanea sativa* | [F1AHA2](https://www.uniprot.org/uniprot/F1AHA2) | 47.1 | 1.00E-05 | Cas s 8 |  |
| 191 | Ara h 9 | *Arachis hypogaea* | [B6CEX8](https://www.uniprot.org/uniprot/B6CEX8) | 41.3 | 6.00E-05 | Ara h 9 | B6CEX8 |
| 192 | Can s 3 | *Cannabis sativa* | [P86838](https://www.uniprot.org/uniprot/P86838) | 75 | 7.00E-05 | Can s 3 |  |
| 193 | Cit l 3 | *Citrus limon* | [P84160](https://www.uniprot.org/uniprot/P84160) | 65 | 4.00E-04 | Cit l 3 | P84160 |

Supplement 2

Input protein sequences Ara h 1 (UniProtID P43238), Ara h 5 (UniProtID D3K177), and Ara h Agglutin (UniProtID P02872) from *Arachis hypogaea* to identify clinically relevant protein allergens using AllerCatPro 2.0.

>Ara h 1 (UniProtID P43238) Arachis hypogaea

MRGRVSPLMLLLGILVLASVSATHAKSSPYQKKTENPCAQRCLQSCQQEPDDLKQKACESRCTKLEYDPRCVYDPRGHTGTTNQRSPPGERTRGRQPGDYDDDRRQPRREEGGRWGPAGPREREREEDWRQPREDWRRPSHQQPRKIRPEGREGEQEWGTPGSHVREETSRNNPFYFPSRRFSTRYGNQNGRIRVLQRFDQRSRQFQNLQNHRIVQIEAKPNTLVLPKHADADNILVIQQGQATVTVANGNNRKSFNLDEGHALRIPSGFISYILNRHDNQNLRVAKISMPVNTPGQFEDFFPASSRDQSSYLQGFSRNTLEAAFNAEFNEIRRVLLEENAGGEQEERGQRRWSTRSSENNEGVIVKVSKEHVEELTKHAKSVSKKGSEEEGDITNPINLREGEPDLSNNFGKLFEVKPDKKNPQLQDLDMMLTCVEIKEGALMLPHFNSKAMVIVVVNKGTGNLELVAVRKEQQQRGRREEEEDEDEEEEGSNREVRRYTARLKEGDVFIMPAAHPVAINASSELHLLGFGINAENNHRIFLAGDKDNVIDQIEKQAKDLAFPGSGEQVEKLIKNQKESHFVSARPQSQSQSPSSPEKESPEKEDQEEENQGGKGPLLSILKAFN

>Ara h 5 (UniProtID D3K177) Arachis hypogaea

MSWQTYVDNHLLCEIEGNHLSSAAILGQDGSVWAQSSNFPQFKPEEITAIMNDFAEPGSLAPTGLYLGGTKYMVIQGEPGAVIRGKKGPGGVTIKKTNQALIIGIYDEPMTPGQCNMIVERLGDYLIDTGL

>Ara h Agglutin (UniProtID P02872) Arachis hypogaea

MKPFCVFLTFFLLLAASSKKVDSAETVSFNFNSFSEGNPAINFQGDVTVLSNGNIQLTNLNKVNSVGRVLYAMPVRIWSSATGNVASFLTSFSFEMKDIKDYDPADGIIFFIAPEDTQIPAGSIGGGTLGVSDTKGAGHFVGVEFDTYSNSEYNDPPTDHVGIDVNSVDSVKTVPWNSVSGAVVKVTVIYDSSTKTLSVAVTNDNGDITTIAQVVDLKAKLPERVKFGFSASGSLGGRQIHLIRSWSFTSTLITTTRRSIDNNEKKIMNMASA

Supplement 3

Input sequence of MatR (UniProtID C6ZI77) from *Rafflesia arnoldii*, a rare plant endemic to the island of Sumatra in Indonesia, to exemplify the importance of evaluating AllerCatPro 2.0 prediction results in context of information on human exposure, especially for less well characterized protein sequences.

>MatR (UniProtID C6ZI77) Rafflesia arnoldii

DDPKFFYSIHKVFSARRLVGGENAPDSVPHXVLLSALPGNIYLHKLDQEIGRIRHKYEITIVQRIRSFMTGRIDDQEQDGEEASFHAPQGNIALFFGXXIQRKAAFPSLFSSXHTPRKTPRGDQKKPSAPALAAFMNKPSSLLCAALLMESVTLKAELYGRERWAMRDLIQSCKRKGLLIELGGEARLVLRSERRLARKLAPLKTHDFIHLSYARYANDLLLGIVGASELLYEIQKRIAQFLQSGLTGSAGSTTIAARSTVEFLGTVIREVPTTIQFFRELEKRIRVKHRIHITACHLRSAIHSKFRNLGKSIPIKQLTKEMSEKGSLLDGVAETLGTTGVRSPQASVLWGTFQHIRQGSRGISLLHSSGRSKAPSDVQQAVSRSGMSVRLYTPAGLKAAGEGGGAGSISREFPINIEAPIKKILRMLQDRGLISRRRPWPIHVACLTSVSDGDIVNWSAGIAISPL
